# A quest for genetic causes underlying signaling pathways associated with neural tube defects

**DOI:** 10.3389/fped.2023.1126209

**Published:** 2023-05-22

**Authors:** Sunil Rai, Larissa Leydier, Shivani Sharma, Jigar Katwala, Anurag Sahu

**Affiliations:** ^1^ Department of Molecular Biology, Medical University of the Americas, Charlestown, Saint Kitts and Nevis; ^2^ Institute of Medical Sciences, Banaras Hindu University, Varanasi, Uttar Pradesh, India

**Keywords:** neural tube defects (NTDs), gene, signaling pathway, genetic factors, folate

## Abstract

Neural tube defects (NTDs) are serious congenital deformities of the nervous system that occur owing to the failure of normal neural tube closures. Genetic and non-genetic factors contribute to the etiology of neural tube defects in humans, indicating the role of gene-gene and gene-environment interaction in the occurrence and recurrence risk of neural tube defects. Several lines of genetic studies on humans and animals demonstrated the role of aberrant genes in the developmental risk of neural tube defects and also provided an understanding of the cellular and morphological programs that occur during embryonic development. Other studies observed the effects of folate and supplementation of folic acid on neural tube defects. Hence, here we review what is known to date regarding altered genes associated with specific signaling pathways resulting in NTDs, as well as highlight the role of various genetic, and non-genetic factors and their interactions that contribute to NTDs. Additionally, we also shine a light on the role of folate and cell adhesion molecules (CAMs) in neural tube defects.

## Background

**Figure 1:**
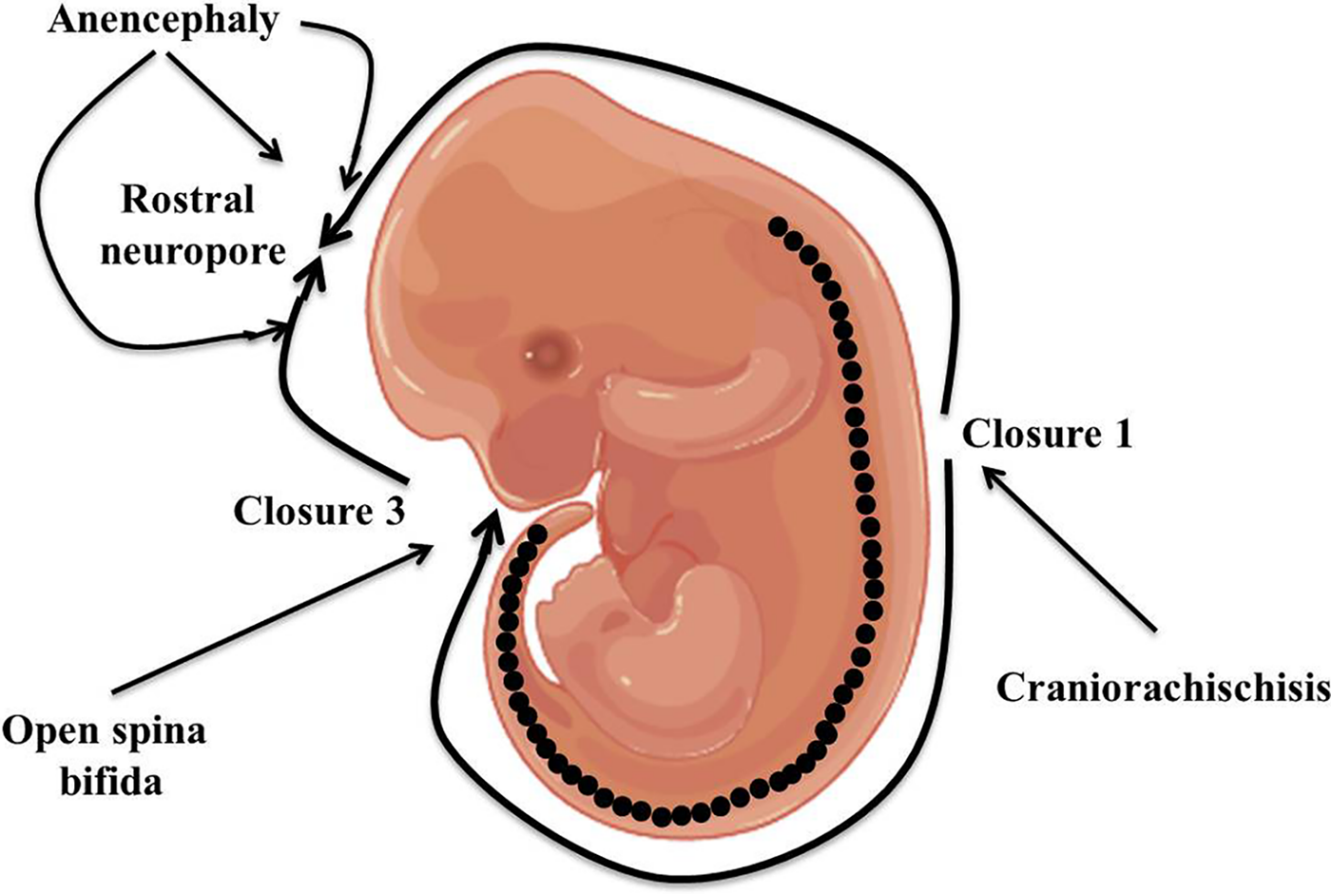
Diagrammatic representation of closure of the neural tube and the origin point for open neural tube defects.

Neural tube defects (NTDs) are the most prevalent serious human birth anomalies of the brain and spine that occur during embryogenesis (by the end of the 6th week of pregnancy). NTDs originate owing to the failure of the neurulation process, which represents the failure of the harmonized morphogenetic process involved in neural tube closure (1, 2). NTDs affect ten infants per 1,000 established pregnancies but this figure varies among different populations (3). The highest prevalence of NTDs has been reported in the Chinese population while the lowest prevalence is in Scandinavian countries (4–7). In India, the incidence of NTDs, especially in the northern part of the country, is approximately 7.8 per 1,000 births (8). NTDs are categorized into two kinds: open and closed NTDs. Open and closed NTDs have affected areas that are either exposed to the body surface or covered with skin, respectively. Anencephaly and spina bifida are the two most prevalent types of open NTDs that arise due to the failure of closure of neural tubes at cranial and spinal regions, respectively (Figure 1). Closed NTDs are classified based on the presence (lipomyelomeningocele, lipomyeloschisis, myelocystocele, and meningocele) or absence (caudal regression, dermal sinus, segmental, and spinal dysgenesis) of a subcutaneous mass (1). Studies based on population and family suggested an intricate etiology for NTDs that involves both genetic and environmental factors (9–13). Genetic mechanisms underlying NTDs are extremely complicated and follow multi-factorial inheritance that is regulated by the interaction of many genes and environmental factors (14). Harmonized gene programs are characteristic features of embryonic development, which is essential for normal neural tube formation. Alteration in harmonized gene programs involved in different signaling pathways (BMP, Wnt, Shh, FGF, TGFβ, etc.) culminates in NTDs (15–19). Recently, accumulating studies performed on genetic models and patients with NTDs revealed that the deregulation of multiple genes associated with signaling pathways, such as WNT, BMP, SHH, and retinoic acid (RA) signaling, culminates in NTDs (20–25). Hence, here we review what is known to date regarding altered genes associated with specific signaling pathways resulting in NTDs, as well as highlight the role of various genetic and non-genetic factors, and their interaction that contribute to NTDs. Additionally, we also shine a light on the role of folate and cell adhesion molecules (CAMs) in neural tube defects.

## Etiology—neural tube defects

The formation of the neural tube is a multistep, zipper-like, and discontinuous process regulated by multiple genes and is affected by environmental factors of the host. It involves gene-nutrients, gene-environment, and gene-gene interactions. Animal and clinical studies in the last five decades have recognized the etiology of neural tube defects, which comprise genetic, epigenetic, environmental, and nutritional factors (Table 1) (26–28). It is well-reported that genetic factors are responsible for 70% of the variance of neural tube defects (29).

(i) **Non-genetic factors:** Non-genetic factors indirectly affect the process of neural tube formation by modulating the gene functions that are discussed as follows:
  a) **Nutritional factors:** The majority of congenital birth deformities occur especially in families with lower socioeconomic statuses, which leads to **the** evaluation of **the** involvement of nutritional factors in neural tube defects. It is well-reported that maternal nutrition plays a crucial role during the normal growth and development of the fetus. It can also affect the capacity of fertilization and the quality of gametes. An abundance of studies showed that polymorphism in folate metabolizing genes (MTRR and MTHFR) is associated with increased chances of non-disjunction (30–32). A low level of B-vitamin folate was noticed in fetuses with neural tube defects (33), inducing a clinical trial of folic acid supplementation to reduce the population burden of neural tube defects. A multi-centric randomized controlled trial showed that supplementation of folic acid mitigates (4mg/day) the occurrence of neural tube defects (34). Several other clinical trials confirmed the reduction of neural tube defects after the uptake of folic acid (35–37). Numerous previous studies suggested that the supplementation of folic acid reduced the occurrence of neural tube defects by 50%–70% (37, 38). Sub-optimal levels of folate may trigger neural tube defects in individuals who carry genetic mutations in the pax3 gene (39). Numerous experimental and clinical studies showed that alteration in purine and thymidylate biosynthesis is linked with the development of neural tube defects (40–42). A study conducted on curly tail mice showed that myo-inositol prevents cranial and spinal neural tube defects (43). Clinical studies reported no recurrence of neural tube defects in neonates with a combination of inositol and folic acid (44, 45). Previous studies demonstrated the relationship between Zn and neural tube defects (46, 47). The status of folate may also affect the gene expression associated with neurodevelopment as they influence histone modification and DNA methylation (48). Folate of maternal plasma affects the differential methylation of DNA in the newborn, leading to alterations of gene expression that eventually culminate in neural tube defects (49). Clinical data demonstrated that GNAS imprinting plays a crucial role in the regulation of folic acid metabolism during embryogenesis and that alteration in GNAS imprinting clusters leads to neural tube defects (50). Folate deficiency promotes the monoubiquitination of H2A histone, resulting in decreased expression of genes (Gata4, Cdx2, Pax6, and Nes) associated with neural tube closures in the embryonic stem cells of mice (51). However, the exact mechanisms underlying folate-deficiency-induced neural tube defects are still not known.
  b) **Hyperthermia:** Elevated body temperature (>40°C) during the pregnancy is nominated as maternal hyperthermia and may happen because of fever, hot water baths, and the usage of saunas and hot tubs, causing developmental deformities (52, 53). *In vivo* and *in vitro* studies on different animal models showed that the neural tube is highly sensitive to elevated temperature (32). Hyperthermia influences multiple developmental processes such as cell differentiation, migration, apoptosis, and proliferation (32, 54). The impacts of heat stress on embryogenesis depend on the duration and dose of the heat exposure, strain, species, and stage of embryonic development (55, 56). Clinical case and animal studies reported the teratogenic and mutagenic effects of hyperthermia (56–60). Significant upregulation of expression of Cx43 mRNA (connexin 43) was observed in neural tubes, especially in heat-treated groups in contrast to the control, indicating a relationship between upregulated Cx43 mRNA and neural tube defects (53). Exposure to the influenza virus during the first trimester induces the risk of the development of neural tube defects (58). Nine case report studies clearly showed a clear relationship between maternal exposure to hyperthermia and elevated risk of neural tube defects (57). A study performed in California, United States, also observed similar effects of febrile illness and maternal fever on neural tube cases (61). A cohort study conducted on 23,491 women reported the association between maternal exposure to hyperthermia through various sources (hot water baths, hot tubs, fever, and sauna) and the risk of the development of neural tube defects (62). A combination of different sources of hyperthermia increased the risk of neural tube defects (59). A comparative study conducted on the population of the Texas-Mexico border showed that maternal exposure to hyperthermia during the first trimester enhanced the relative risk of development of neural tube defects by 3.6% (63). However, recent studies on pregnant women do not observe any fetal abnormalities after COVID-19 vaccination (64–68).
  c) **Pesticides:** The population explosion increased the demand for the development of novel approaches to enhancing agricultural production to fulfill the increased demand, and these new methods were highly dependent on the utilization of pesticides. Continued and injudicious use of pesticides increased its residues in fruits, vegetables, cattle milk, cattle meat, food, and water, enhancing the risk of exposure to pregnant women. Several studies demonstrated the negative medical side effects caused by an enhanced and indiscriminate use of pesticides (69, 70). Several lines of evidence reported that pesticides contribute to significant developmental and reproductive disorders with carcinogenic and teratogenic capabilities (69, 71, 72). Several previous experimental and case reports indicated a connection between congenital disorders and pesticide exposure (69–71, 73, 74). A study performed in Washington, USA, showed the increased risk of development of neural tube defects post pesticide exposure (75). A case-control study demonstrated that pesticide exposure induced the developmental risk of neural tube defects (76). Another study performed on a case group found that there were 2 times greater chances of neural tube defects affecting pregnancies in individuals who were living 0.25 miles from agricultural fields or using pesticides at home (70). A population-based case-control study showed a marginal or zero developmental risk of neural tube defects post-pesticide exposure (63). Another study proved that other confounding factors (folate deficiency and low level of vitamin B12) may increase the developmental risk of neural tube defects on pesticide exposure. A systematic review showed that, due to the heavy usage of pesticides, the occurrence of neural tube defects in neonates is more prominent in developing countries such as those in the African continent (72). A case report study based on a questionnaire as directed by the WHO found an increased incidence of neural tube defects post-maternal exposure to pesticides (71). A study conducted on agriculture workers observed a higher incidence of congenital deformities in neonates (77).
  d) **Arsenic (As):** Globally, the level of arsenic has increased due to metalworking industries, the combustion of coal, and the production of pesticides, resulting in contamination of inorganic arsenic in air, water, and soil. Approximately 95% of Arsenic absorption among Europeans is due to the consumption of arsenic-contaminated foods (48). Several lines of evidence reported the teratogenic and toxic properties of arsenic and found that it is an utmost risk for the development of neural tube defects (78–80). Numerous past studies reported that arsenic disrupts the placental structures, resulting in the disruption of the transport of nutrients and molecules (79–82). Animal and human studies showed that Arsenic induces neural tube defects because it triggers epigenetic alteration and gene mutation (80, 81). A case-control study based on GWAS recognized the 14 single nucleotide polymorphisms (SNP) expressed in neural tube defects pregnancies post arsenic toxicity (80). DNA methylation is a crucial process during the developmental period and is influenced by arsenic poisoning. Studies based on arsenic poisoning showed that it inhibits DNA methylation by reducing the activity of DNA methylase (1 and 3b) and S-adenosyl methionine (SAM) (48, 83, 84). Folate interacts with arsenic resulting in a reduction of arsenic in blood as well as an extensive efflux of folate (79). A case-control study conducted in Bangladesh showed that high efflux of folate owing to the interaction with arsenic increases the risk of neural tube defects (80). A case study that included 49 mothers and their neonates showed a clear relationship between arsenic levels in the environment and the placenta (82). The research also reported increased levels of lipid peroxidase and reduced glutathione in the blood and placenta, leading to increased oxidative damage. The states of Assam and Uttar Pradesh, in India, were dependent on rice and consumption of groundwater and developed arsenic belts between the regions, as is indicated by the high incidence of neural tube defects in these regions (78).
  e) **Polyaromatic hydrocarbons (PAHs):** PAHs are environmental pollutants that arise through anthropogenic activities particularly owing to the incomplete combustion of wood, oil, coal, and petrol (85). PAHs have several medical side effects including enhanced risk of neural tube defects. A study conducted in the rural population of Shanxi province, China found that women with coal exposure (IAPCC) had a 60% enhanced risk of having newborns with neural tube defects in contrast to women without IAPCC exposure (86). Maternal occupational exposure to PAHs was found to enhance the risk of neonates with spina bifida amongst women with underweight or normal weight (87). Higher concentration of PAHs has been reported in the placenta in cases of neural tube defects (88). A woman with an elevated concentration in the serum was found to be associated with a high risk of neural tube defects in neonates (89). However, the molecular mechanisms underlying PAHs-induced neural tube defects are not well known. On the contrary, a recent study by Huang et al. (90), showed that reduced global DNA hypo-methylation could be one of the possible mechanisms underlying the increased risk of neural tube defects induced by PAHs.
  f) **Antibiotics:** Antibiotics are employed to treat bacterial infections, such as acute cystitis and bacteriuria, experienced by pregnant women. Past studies revealed that antibiotics cause functional and physical deformities in the fetus or human embryo (91, 92). A study found that antibiotics prescribed for the management of urinary tract infections (UTIs) were linked with neural tube defects in neonates (93). Epidemiological reports observed that the trimethoprim drug increased the risk of both childbirth deformities and miscarriage (93–95). A population-based case-control study noted the association between the use of antibiotics during the first trimester and birth deformities in neonates (91). One study discovered the association between nitrofurantoin exposure during the first trimester and enhanced risk of birth defects in neonates (96). A population-based cohort study showed that gestational exposure to nitrofurantoin is marginally linked with developmental malformations (97). Antibiotics such as non-steroid anti-inflammatory drugs (NSAID), paracetamol, and opioids are prevalent drugs employed for the management of pain. Concurrent usage of opioid drugs and NSAIDs for the management of pain was found to be associated with a higher incidence of spina bifida in contrast to singular drug-mediated pain medication (98). Some of the studies also found similar outcomes with the usage of opioid drugs (99, 100). A study conducted in the USA reported the connection between the usage of anti-epileptic drugs and the incidence of cleft palate and spina bifida (101).
  g) **Trace Elements—Neural Tube Defects:** Trace elements are chemical compounds in organisms that are required in minuscule amounts for physiological functions. Trace elements are divided into two groups: Essential trace elements (ETEs) and Non-essential trace elements. ETEs include Zn, Mn, Co, Mo, Fe, and Se; these trace elements play a key role in fetal and maternal health during pregnancy (102–104). Studies showed that ETEs are involved in cell function and differentiation, suggesting that ETEs play a key role in multiple physiological and cellular functions. Therefore, an alteration in the homeostasis of ETEs during pregnancy may lead to birth defects (105). Insufficient dietary intake of Fe is linked with a higher risk of spina bifida (106). Higher concentrations of Mn in maternal blood during pregnancy significantly increase the risk of NTDs (107). Studies have demonstrated that low selenium levels in maternal plasma and serum are associated with an enhanced risk of NTDs (108, 109). Several lines of evidence have demonstrated that lower concentrations of Zn in maternal serum and scalp hair are linked with increased risk for NTDs in offspring (110–112). However, some studies found that a higher concentration of Zn in maternal hair during the peri-conceptional period and nails during the third trimester is linked with elevated risk for NTDs (113, 114). Previous studies also observed the association between the level of Mo and Co and enhanced risk of NTDs in offspring (115, 116). Alkaline earth metals such as Ba, Th, and Cs also cause neural tube defects in children (117–119). Maternal exposure to Ba during the embryonic period leads to the development of NTDs in offspring (117). A case-control study has demonstrated the association between NTDs and Th levels (118). Another study led by Pi et al. (119), observed the association between Cs level and increased risk of NTDs (119).
(ii) **Genetic factors:** Neural tube defects are multi-factorial in origin (2, 120). Epidemiological evidence on humans showed that the genetic basis for neural tube defects is acquired from the positive concordance of neural tube defects from monozygotic twins in contrast to di-zygotic twins (121, 122). In mice, more than 400 genes are involved in the closure of the neural tube (123, 124), and approximately 191 NTD candidate genes are found in NTD fetuses (125). Although defects in neural tube closure occur more familiarly after one neural tube defect-affected pregnancy (recurrence rate is 1 in 20), the recurrence rate of neural tube defects does not exceed 10% even after two neural tube defect-affected pregnancies. These recurrence risks strongly indicate the involvement of multiple genes in neural tube defects. The risk of recurrence and pattern of inheritance of neural tube defects in the multiplex families do not follow the Mendelian law of inheritance (126). Some studies showed that both sex-influenced and maternal genetic factors contribute to the developmental risk of neural tube defects (127, 128). The estimated heritability rate in neural tube defects is approximately 60%, especially when multiple susceptible genes are involved (12). Animal models are very crucial in understanding the role of candidate genes in the development of neural tube defects because the process of neurulation is very similar in humans and mice. Several gene ablations that were responsible for neural tube defects in mice models echoed the few cases of neural tube defects observed in humans, such as Pax3 (paired box-3 protein) and Lrp6 (low-density lipoprotein receptor-related protein-6) (129, 130). Apart from animal models, next-generation sequencing (NGS) shines a new light on underlying molecular insight of genetic risk factors for neural tube defects that includes whole exome sequencing (WES), target panel sequencing (TPS), and whole genome sequencing (WGS). One research recognized the homozygous missense genetic ablation in the TRIM36 gene by using WGS, which is responsible for autosomal recessive anencephaly, particularly in Indian families (131). Another study identified the *de novo* damaging variants of anencephaly through WES (132). Ishida et al. (133), identified the 397 damaging variants of anencephaly cases through TPS, in which 21 variants out of the 397 had not been previously reported. A recent study used WGS to reveal the genetic mutation in non-coding regions that contributes to neural tube defects (134). Several studies have demonstrated the association between mutations in epigenetic regulators and enhanced risk of NTDs (28, 120, 135).
(iii) **Epigenetic Factors:** An epigenetic mechanism of gene regulation makes stable phenotypic changes without any change in the nucleotide sequence of DNA. Epigenetic regulators play a pivotal role in global gene regulation. Several studies have demonstrated the association between mutations in epigenetic regulators and enhanced risk of NTDs (28, 120, 135). Alterations in DNA methylation, chromatin remodeling, and histone modification may lead to an increased risk of NTDs (28, 136). It has been shown that DNA methylase 3A (DNMT3A) and DNMT3B are responsible for demethylating and remethylating the majority of the embryonic genome except for the imprinting region, while DNMT1 maintains the methylation pattern (137). Mice deficient in DNMT3A and DNMT3B had an increased risk of NTDs, indicating that appropriate remethylation is essential prior to implantation (138). Extensive studies have demonstrated the association of folate one-carbon metabolism with an elevated risk of NTDs owing to diminished methylation (139, 140). A study conducted on splotch embryos showed that enhanced methylation of H3K27 in neural crest cells leads to an increased risk of NTDs (141). Knockout mice of p300 (histone acetyltransferase enzyme) exhibited cranial NTDs, suggesting that it is essential for the closure of the neural tube (142). Studies have found that mutations in Gcn5 and Cited2 disrupt HAT activity and elevate the risk of NTDs (143, 144). Pharmacological inhibitors such as valproic acid and trichostatin-A demolish the regulation of acetylation that causes NTDs (145, 146). Mutations in histone deacetylase (*hdac4* and *sirt1*) cause cranial NTDs (147, 148). Mutations in several chromatin remodeling enzymes are associated with NTDs (121, 149). Several studies showed that mutation in SMARCC1, CERCR2, BRD2, and SMARCA4 is linked with an enhanced risk of NTDs (150–153).

**Table 1:** Factors linked with developmental risk of neural tube defects.

Factors	Affected Genes	Effects
**1. Non-genetic factors**		
Nutritional Factors	Decreased expression of Cdx2,Gata4, Nes and Pax6	Neural tube defects
Hyperthermia	Aberrant expression of Cx43 mRNA	Neural tube defects
Pesticides		Enhanced the risk of NTDs
Arsenic (As)	Induce the perturbation in DNA Methylation	Neural tube defects
Polyaromatic aromatic hydrocarbon (PAH)		Enhanced the risk of NTDs
Antibiotics		Anencephaly –Antibiotics Spina Bifida -NSAIDs, Opiods and anti-epilectics
2. Genetic Factors	Aberrant expression of Lrp6 and Pax3	Neural tube defects
	Mutation in TRIM36	Anencephaly
	Mutation in BRCA1	Neural tube defects
	Mutation in CFL1	Neural tube defects
	Mutation in CITED2	Neural tube defects
	Mutation in PDGFRA	Neural tube defects
	Mutation in PRKCA & B	Neural tube defects
	Mutation in TXN2	Neural tube defects
	Mutation in TP53	Neural tube defects
	Mutation in ZIC1/2/3	Neural tube defects

## Signaling pathways—neural tube defects

Neurulation occurs in two phases in mice and humans (primary and secondary) from embryonic day 8.5 to 10.5 **(**day 22–23 and 26–30 of gestation in humans**)** (154). The neural tube is an embryonic precursor that develops later into the spinal cord and brain through fine-tuned coordination of multiple signaling pathways, including planar cell polarity (PCP) signaling, sonic hedgehog (Shh) signaling, bone morphogenetic protein (BMP) signaling, inositol metabolism, retinoid signaling, canonical Wnt signaling, fibroblast growth factor (FGF) signaling, tumor growth factor (TGF-β) signaling, Notch signaling, receptor tyrosine kinase-like orphan receptor (ROR) signaling, and folate-methionine metabolic signaling pathway, during the time window that is required for closure of the neural tube (155). Genes associated with these signaling pathways are involved in epigenetic modifications (acetylation and methylation), organization of chromatin, regulation of the cell cycle, and actin cytoskeleton (156). The perturbation in genes and cross-talk between signaling pathways leads to the pathogenesis of neural tube defects (120) (Table 2), which are discussed as follows:

(i) **Planar cell polarity (PCP) signaling pathway:** PCP signaling is required for the closure of the boundary between the cervical and hindbrain; hence, it is nominated as a planar signaling pathway owing to its involvement in the coordinated polarized orientation of cells. Planar cell polarity was originally described in a *Drosophila* model as a signaling cascade that mediates its action without the requirement of β-catenin; so-called as a non-canonical Wnt signaling pathway and required for specification of plane polarity in epithelia, including compound eye and wing (156). PCP signaling pathway is highly conserved in vertebrates and involved in various developmental processes such as cellular and tissue polarity during morphogenesis and harmonized orientation of hair cells of the inner ear (156–159). Positioning cloning of *Vangl2* in loop-tailed mutant mice that exhibited severe forms of neural tube defects (craniorachischisis) was the first evidence that shed a light on the role of PCP signaling pathway in the pathogenesis of neural tube defects (160, 161). Experimental studies showed that a double mutant of *Fzd* (Frizzled)-3 and -6 *Dvl* (disheveled)-1 and -2 protein contributes to the pathogenesis of craniorachischisis (162, 163). Several lines of experimental studies linked the other PCP-related genes (*Srb1* and *Ptk7*) with the development of severe neural tube defects (161, 164). Genetic ablation in Sec24b contributes to the pathogenesis of neural tube defects (165). They also reported that the mutant form of *Sec24b* significantly enhances the prevalence of spina bifida by interacting with the LoF (loss of function) *Vangl2* allele. Mutational studies showed that mutation of Sec24b, Ptk7, or Sdc4 contributes to craniorachischisis in combination with a heterozygous allele of Vangl2^Lp^/^+^ (161, 164, 166). Combination of Vangl2^Lp^/^+^ with genes (Fzd2^+/−^, Fzd1^+/−^, and Dvl3^+/−^) of the Wnt signaling pathway contributes to the risk of exencephaly (167, 168). Some other studies showed that PCP effector genes (*Fuz* or *Intu*) are also responsible for exencephaly (169–171). A mutational study on mice showed that genetic ablation in *Smurf1/2* leads to PCP-related neural defects (172). The digenic combination of double knockout *Vangl2* with *Cthrc1* or *cordonbleu^C101^* contributes to exencephaly (173, 174). Experimental mutational studies on mice demonstrated that Ptk7 (PCP genes) with *Grh13* (non-PCP genes) develops spina bifida (164, 175) while with *Cthrc1* develops exencephaly (175). A mutational study performed on mice models demonstrated the role of *Celsr1*in the pathogenesis of neural tube defects (176). A genetic study performed on a circle-tail mouse found that dysfunction of the *Scrb1* (Scribb) gene contributes to neural tube defects (177). The candidate genes identified in the animal model provide the rationale for the recognition of orthologous genes involved in human neural tube defects. The Orthologue of *Vangl2* was the first human gene of PCP signaling implicated in neural tube defects. A study conducted with Italian patients analyzed the role of *Vangl2* and its paralogue *Vangl* (178) and reported on the three variants of *Vangl: p.Val239Ile* and *p.Arg274Gln* were involved in familial neural tube defects while *p.Met328Thr* was involved in sporadic cases of the disease. The *p.Val239Ile* mutation inhibited the interaction between *Dvl* proteins and *Vangl1.* Several clinical studies demonstrated the role of the *Vangl1* gene in human neural tube defects (16, 179–183). Embryo with double heterozygous mutation of *Vangl2^Lp^* and *Ptk7^XST87^* exhibited the development of spina bifida (184). Genetic studies also implicated the role of various genes (*CELSR1–3, PRICKLE1, FZD6, LRP6,* and *SCRIB*) in human neural tube defects (185–190). A missense mutation in ANKRD6 alters the reciprocal antagonism mechanisms between both Wnt signaling pathways involved in neurulation, resulting in NTDs (187). LRP6 is another candidate gene that encodes DIVESIN and functions as an antagonist on both Wnt signaling pathways (188). Genetic ablation of LRP6 leads to spina bifida (129). In another study, mutations in WDR34 impaired the PCP signaling pathway, increasing the risk of NTDs (191).
(ii) **Canonical Wnt signaling pathway:** Wnt/β-catenin signaling pathway is involved in anterior-posterior patterning during embryonic development and any perturbation in this process culminates in neural defects. Wnt signaling is also involved in the activation of the PCP signaling pathway through stimulation of Rho-dependent kinase (192). Altered expression of the Wnt signaling pathway leads to impairment in anterior-posterior patterning, resulting in NTDs (193). Genetic alteration in β*-catenin* with *Pax3* contributes to spinal neural tube defects (194). Recently, one study conducted on a mouse model suggested that abnormal expression of Gcm1protein linked with the Wnt signaling pathway leads to neural tube defects (192). Habert et al. (195) observed the burden of deleterious SNPs associated with canonical Wnt signaling genes in patients with myelomeningocele. Several experimental studies reported the molecular switches, such as *Ptk7* and *Lrp6,* that regulated the involvement of the Wnt signaling pathway (canonical and non-canonical) in the closure of neural tube defects (21, 196, 197). Some studies showed that *Ptk7* mutation abrogates the targets of the canonical Wnt signaling pathway, resulting in failure of neural tube closure (198, 199). Another study conducted on animal models showed that *Ptk7* and *Lrp6* alter the activity of the canonical signaling pathway, resulting in neural tube defects (199). Exome sequencing analysis showed that mutations in ten Wnt genes are prominent among Mexican-American patients with myelomeningocele (21).
(iii) **Sonic hedgehog (Shh) signaling pathway:** Shh signaling pathway plays a crucial role in patterning, growth, and morphogenesis during embryonic development. It regulates the patterning of the ventral neural tube and its extension into the brain regions (200). Several lines of studies showed that genetic ablation in Ptc1 (patched) contributes to the failure of neural tube closure (201, 202). Negative mutation in Shh signaling inhibitory genes gives rise to neural tube defects (201–203). Some of the studies suggested that the overexpression of Smo and Shh proteins of Shh signaling may lead to the failure of neural tube closure (201, 202). Studies based on a knockout mouse model showed that Fkbp8 (FK506 binding protein-8) mutation leads to the development of spina bifida (204, 205). Mutation in many other genes of the Shh signaling pathway contributes to exencephaly (206–218). Some studies also implicated the mutation in the genes (Ptch1, Rab23, and Tulp3) of the Shh signaling pathway in the development of spina bifida and CRN (209–212, 217, 218). Another study showed that mutation in protein required for the function of cilia leads to impaired Shh signaling pathway, culminating with neural tube defects (17). Accumulating evidence on humans also showed that genetic ablation in the Shh signaling gene leads to the development of neural tube defects (219–221). Genetic ablation of the WDR34 gene impaired the Shh signaling pathway resulting in exencephaly (191).
(iv) **BMP (bone morphogenetic protein) signaling pathway:** BMPs are members of the TGF-β superfamily that acts as a morphogen, involved in the development, patterning, and function of the nervous system. It is needed for the development of dorsal neural tubes, especially for stimulation of dorsal neurons and neural crest cells (NCC) prior to neurulation. Animal and human studies showed that knockout mice with *BMP4* and *NOG* (noggin) lead to neural tube defects (15, 19, 222–225). Evaluation of *BMP4* and *NOG* showed that the genetic alteration in both genes resulted in neural tube defects in humans (222). Evidence from the knockout mouse model showed that mutation in *Noggin* culminates in exencephaly and spina bifida (15). Studies on genetic mouse models observed that *BMP2* mutation culminates in premature as well as exaggerated bending of caudal neuropore and various cranial deformities (225, 226). Genetic studies performed on mouse models showed that double knockout of *Bmpr1A* and *Bmpr1B* leads to the development of holoprosencephaly (227–229). Genetic analysis based on the double mutant of *Bmpr1A* and *Bmpr1B* showed the existence of two kinds of holoprosencephaly (227). Embryo with *Zic2* mutation leads to the development of spina bifida owing to the absence of DLHP required for closure of the neural tube in the lower region spinal cord (226).
(v) **Retinoid signaling pathway:** Retinoic acid, a derivative of vitamin-A, is crucial for the patterning of the spinal cord and hindbrain (229). An imbalance in the level of vitamin-A and retinoic acid has been implicated in birth defects including neural tube defects (230–232). Negative mutation in *Raldh2* (key enzymes involved in retinoic acid synthesis), *Cyp26a1* (key metabolizing enzyme), and *retinoic receptors α* and *γ* contributes to neural tube defects (233–235). A case-control study identified the association of variants of *Raldh1A2, Cyp26A1,* and *CRABP1*retinoic genes and neural tube defects in humans (230). Experimental studies on mouse models have shown that overexpression of retinoic acid leads to neural tube defects (236, 237). A recent study found that treatment of neural crest cells (NSCs) with all-trans-retinoic acid culminates in neural tube deformities (238).
(vi) **Notch signaling pathway:** The notch signaling pathway regulates the proliferation and differentiation of NSCs (neural crest cells) during embryonic development. These NSCs are required for the normal closure of the neural tube (2) and dysregulation of proliferation, migration, and differentiation of NSCs leads to brain anomalies (239, 240). Previous Studies observed that mutation in the genes *Hes1, Hes3,* and *RBP-Jκ* of the Notch signaling pathway contributes to neural tube anomalies (121, 149). A recent study observed that abnormal expression of N1 (Notch1) enforces the occurrence of neural tube deformities (238). A study conducted on embryonic stem cells showed that the double mutant embryo of *CSL* (CBF-1/Suppressor of hairless/Lag-1) displays the phenotypes of neural tube defects (241). Overexpression of *Notch3* in the nervous system of mice has been implicated in exencephaly (242).

**Table 2:** Genes of signaling pathways linked with developmental risk of neural tube defects

Signalling Pathways	Affected Genes	Outcomes
1. Planar cell polarity (PCP)	Fzd-3& 6, Dvl-2 & 3	Craniorachischisis
	Srb1 & Ptk7	Neural tube defects
	Sec42b with Vangl2	Spina bifida
	Fuz or Intu	Exencephaly
	Vangl2 with Cthrc1	Exencephaly
	Ptk7 with Grh13	Spina bifida
	Celsr1 and Scrb1	Neural tube defects
2. Canonical Wnt	*Β*-Catenin with Pax3	Spinal NTDs
	Ptk7 and Lrp6	Neural tube defects
3. Sonic hedgehog (Shh)	Ptc1	Neural tube defects
	Smo and Shh	Neural tube defects
	Fkbp8	Spina bifida
	Ptch1, Rab23 and Tulp3	Spina bifida and Craniorachischisis
4. Bone morphogenic protein (BMP)	BMP4 with NOG	Neural tube defects
	Noggin	Exencephaly and Spina bifida
	BMP2	Neural tube defects
	Bmpr1A & Bmpr1B	Holoprosencephaly
	Zic2	Spina bifida
5. Retinoid	Raldh2 and Cyp26a1	Neural tube defects
	Retinoic receptor *α* and *λ*	Neural tube defects
6. Notch	Hes1, Hes3 and RBP-jk	Neural tube defects
	N1 (Notch)	Neural tube defects
	CSL	Neural tube defects
	Notch3	Exencephaly

## Folate–neural tube defects

Folate is a water-soluble vitamin B that plays a crucial role in nucleotide synthesis and methylation pathway required for cellular proliferation and differentiation during embryonic development (Table 3) (19, 27). Several lines of evidence showed that genetic ablation in *FOLR1*, which encodes the protein required for folate transport, culminates in neural tube defects (243–245). However, mutations in *FOLR2* and *RFC* (trans-membrane receptor) did not cause any congenital abnormalities (244, 246). A study led by Barber et al. (247), showed that the development of neural tubes will be delayed if an ample amount of nucleotide is not available in neuroepithelial cells, indicating the crucial role of folate during embryonic development. Experimental research conducted by Flemming et al. (248), on splotch mouse models supports this hypothesis. The authors concluded that mutation in the *Pax3* gene leads to neural tube defects due to a deficiency of dTMP synthesis. Many studies on the splotch mouse model showed that supplementation with folic acid (FA) or thymidine ameliorates neural abnormalities (41, 248). Embryos with a null mutation in the *SHMT1*gene display an exencephaly similar to the one caused by maternal folate dietary deficiency (249, 250). Impairment in *de novo* synthesis of purine has been reported in homozygous knockout mice for the *MTHFD1*gene resulting in neural tube defects (40). However, this observation has not been reported in heterozygous mice for the *MTHFD1* gene. A mouse model with a null mutation in the *Cited2* gene exhibited exencephaly while this effect was reverted by FA supplementation (251). Previous reports have demonstrated that the proper functioning of methylation cycles is required for the normal closure of neural tubes (252, 253). A delay in the normal closure of neural tubes has been observed in chick embryos when the methylation cycle is inhibited by using inhibitors (254). Studies performed on mice showed that the *Axd* and *Amt* mutation contributes to the unresponsiveness to FA supplementation (255, 257). Several lines of experimental studies found that the perturbation in the methylation process owing to folate deficiency leads to a reduction in the normal closure of neural tubes (254, 257, 258). Exposure of an embryo to cycloleucine, an inhibitor of methylation, or Adox (oxidized adenosine), an inhibitor of S-adenosylhomocysteine hydrolase, leads to a delay in the neurulation process (257, 258). Previous reports showed that culturing the mouse embryo with a low concentration of methionine displays the phenotype of neural tube defects (259, 260). A study performed by Bjorklund et al. (261), hypothesized that the post-translation modification of cytoskeleton proteins might be involved in the abnormal closure of neural tubes. One of the studies showed that abnormal modification of actin protein leads to neural tube defects (259).

**Table 3:** Genes of folate-mediated pathway and cell adhesion molecules (CAM) linked with developmental risk of neural tube defects.

	Affected Genes	Effects
1. Folate	FOLR1 (Folate transport)	Neural tube defects
	Pax3 gene (dTMP synthesis)	Neural tube defects
	SHMT1	Exencephaly
	MTHFD1	Neural tube defects
	Cited2	Exencephaly
	Axd and Amt	Unresponsiveness to supplementation of FA
2. Cell adhesion molecules (CAM)	NCAM1	Neural tube defects
	Fat1	Exencephaly
	Integrin-α3/α6 and Perlecan	Neural tube defects
	Laminin-α5	Neural tube defects
	EphrinA5 (EphA5) or EphA7	Neural tube defects
	EphrinB1	Exencephaly

## CAM (cell adhesion molecules)—neural tube defects

CAMs are groups of proteins found at the surface of a cell and are involved in the adhesion of the cell to cell or extracellular matrix; thus acting as a so-called molecular glue. They play a critical role in contact inhibition, cellular growth, and programmed cell death in fully developed animals (262). Apart from this, they also play an essential role in neurulation, cell-cell interaction, axon guidance, and cell migration during neural development (263). Experimental studies on humans and animals showed that mutation in genes associated with CAMs leads to neural tube defects (Table 3) (264–267). A study led by Deak et al. (265) observed the association between SNPs in the NCAM1 (neural-CAM-1) and neural tube defects. Fat1 is a cadherin molecule that is involved in the organization of the cytoskeleton at cell boundaries especially actin polymerization (267). Mutation in the gene of Fat1displays exencephaly, while Fat2 mutation did not cause exencephaly, however, a null mutation in both Fat1 and Fat2 enhanced the frequency of exencephaly in contrast to Fat1 alone (268). Existing literature showed that the mutation in integrins-α3/α6, perlecan, and laminin-α5 genes gives rise to neural tube defects (269–271). A lack of the ephrinA5 or EphA7 gene in mice led to neural tube defects (272–274). Another study reported that the null mutation in EphrinB1 displayed a higher incidence of exencephaly in heterozygous females in contrast to heterozygous males (275).

## Conclusion

Neural tube defects are serious birth defects of the nervous system that occur because of an abnormal closure of the neural tube during embryonic development. Several lines of studies explored the mutated genes responsible for neural tube defects in humans and animals. However, the exact molecular mechanisms underlying neural tube defects are still not known. Advances in whole genome and exome sequencing in the near future may pierce our knowledge of the interactions between teratogens and their effects on the normal closure of neural tubes, leading to the understanding of the molecular mechanisms underlying neural tube defects.

## Author contributions

SR conceptualized the subject, reviewed the literature, and wrote the draft manuscript. LL assisted in the manuscript preparation. SR and AS initiated the topic, designed the figures, and finalized the manuscript. SS and JK contributed in the revision, editing and proofreading of the final manuscript. All authors contributed to the article and approved the submitted version.

## Conflict of interest

The authors declare that the research was conducted in the absence of any commercial or financial relationships that could be construed as a potential conflict of interest.
